# Thermophilic anaerobic digestion of polylactic acid, polyethylene and polypropylene microplastics: effect of inoculum-substrate ratio and microbiome

**DOI:** 10.1007/s10532-025-10186-6

**Published:** 2025-09-30

**Authors:** Mahesh Mohan, Zain Ul Abedien, Prasad Kaparaju

**Affiliations:** 1 School of Engineering and Built Environment, Griffith University, Queensland, Australia; 2 Australian Institute for Microbiology & Infection, University of Technology Sydney, Ultimo, Australia

**Keywords:** Anaerobic digestion, Microplastics, Microbiome, Biodegradation

## Abstract

Microplastics (MPs) generated from major plastic polymers have impacted the environment and formulation of an end-of-life scenario is a need of the hour. In the current study, the effects of inoculum to substrate ratios (ISR) 2, 4 and 6 on the MPs from polyethylene (PE), polypropylene (PP) and polylactic acid (PLA) under thermophilic and mesophilic anaerobic digestion (AD) conditions was studied. The results indicated thermophilic AD to be a prospective method for PLA degradation with a maximum cumulative biogas production of 894.08 NmL/gVS_added_ at ISR4 and 89.62% of volatile fatty acids (VFA) was utilised during 148 days of incubation. However, the thermophilic AD of PP and PE was observed to be highly inefficient with a maximum biogas production of 111.64 and 47.48 NmL/gVS_added_ and also resulted in VFA accumulation. Under mesophilic AD conditions, PLA degradation was highly inefficient due to long hydrolysis time, whilst inhibition was noticed with both PP and PE. The microbiological study revealed the abundance of Firmicutes and Synergistota*,* genus D8A-2, *Thermovirga* and Candidatus Caldatribacterium during thermophilic AD of PLA. An abundance of *Methanothermobacter* indicated hydrogenotrophic methane production as the major pathway for methanogenesis during thermophilic AD of MPs. An abundance of PWY-3781 associated with detoxification of reactive oxygen species was observed in the AD of PP and PE. Overall, the study provided insight into the prospects for improving thermophilic AD for PLA.

**Supplementary Information:**

The online version contains supplementary material available at 10.1007/s10532-025-10186-6.

## Introduction

Plastics as an integral commodity replaced glass, timber, paper and metal, due to its low density, electrical conductivity, corrosion resistance and inexpensiveness. Multitudes of polymer types and its subgroups with varying density, mixtures and blends, majorly belonging to polyethylene (PE), polypropylene (PP), polyethylene terephthalate (PET), polyvinyl chloride (PVC), polystyrene (PS) are commonly available. Its extensive utilization of in everyday lives has resulted in accumulation in the environment as waste. An estimate of 220 million tons plastic wastes was generated worldwide in the year 2024, (Sousa 2024) with an expected rise to 1014 million tons by 2060 (OECD 2022). Plastic wastes are mostly generated as the result of improper disposal, followed by its subsequent accumulation in the environment, causing serious environmental impacts due to its persistence and resistance against degradation (Frias,Nash 2019). The high molecular weight and hydrophobicity, absence of susceptible functional groups, presence of antioxidants, additives and stabilizers, render them the property of impermeability and recalcitrance, which is highly desirable for its use while making them highly resistant to degradation (Chamas et al. 2020).

The plastic wastes exposed to the environment are subjected to weathering and transformation through physical–chemical reactions leading to fragmentation and formation of microplastics (MPs) posing special threat to environment and human health (Zhang et al. 2021). Microplastics, considered to be of 5 mm to 0.1 µm size (Crawford,Quinn 2017) were considered to be a greater threat due to its capacity to enter cells and tissues, as well as modulate toxicity (Triebskorn et al. 2019). The reduced molecular weight and large surface area enhanced its biodegradation through increased microbial colonization (Palmisano,Pettigrew 1992). Microplastics hence served as a niche for biofilms of hydrocarbonoclastic bacteria, Desulfovibrionales and Hyphomonadaceae, indicating possibilities of microbe-mediated degradation through enzyme secretion and subsequent depolymerization (Chai et al. 2020; Kaur et al. 2022; Ogonowski et al. 2018; Othman et al. 2021). While majority of studies are conducted on aerobic degradation of MPs, comparatively less studies are conducted on MPs in anaerobic digestion (AD) (Zhang et al. 2024d), indicating a need to formulate a strategy to degrade major plastic polymers using AD.

AD process is considered optimal when it achieves its peak and stable biomethane production (Lohani,Havukainen 2018) and is directly influenced by factors such as pH, temperature, C/N ratio, organic loading rate (ORT), hydraulic retention time (HRT), toxicity, trace elements and nature of substrates. Thermophilic conditions are generally favourable for acidogenesis, while mesophilic conditions suitable for methanogenesis (Induchoodan et al. 2022). AD involves complex fermentation processes and metabolic pathways driven by a characteristic microbiome (DeCola et al. 2024), highlighting the potential role of hydrolytic bacteria for MP degradation. Anaerobic sludge has also been reported as a source of multiple, unexplored methanogens that contribute to substrate digestion and provide microbiome with potential for developing tailored processes (De Vrieze 2020; Narihiro,Sekiguchi 2007). AD process has been recognised as a promising method for plastic degradation because it allows control over temperature, pH, moisture content, and inoculum, with thermophilic conditions being particularly more conducive for plastic degradation (Quecholac-Pina et al. 2020). Apart from these, AD offers an enclosed system for degradation which helps in monitoring possible degradation intermediates and products that needs to be monitored before its release into the environment. It was also reported to be suitable for the management of biodegradable plastics, with research prospects for standardizing the procedure for its implementation on a practical scale (Cazaudehore et al. 2022a).

The AD of different plastics showed disparity in the degradation kinetics, microbiome and degradation rate and future prospects, with biodegradable plastics including PLA leaning towards better degradation rate compared to petroleum-based plastics (Gómez,Michel Jr 2013; Nachod et al. 2021). The current study was conducted to analyse the potential of using AD for the management of MPs derived from polyethylene (PE), polypropylene (PP) and polylactic acid (PLA).The former two petroleum-based plastics were selected as they were reported to be the majorly used resin type (Kyle O’Farrell 2022). Polylactic acid, a bioplastic claimed to be biodegradable is widely used was not certified under Australia/New Zealand (AS/NZ) certified compostable plastics (Kyle O’Farrell 2022). Also, the a specific surface degradation rate study on PLA indicated it to have a similar value as that of high-density polyethylene (HDPE) in marine ecosystem, while it showed 20 times faster degradation on terrestrial ecosystems (Chamas et al. 2020), indicating the relevance to formulate an efficient management strategy. PLA, PP and PE being the major polymers currently being used in the market was studied for its mesophilic and thermophilic AD. This study is the first to systematically investigate the mesophilic and thermophilic anaerobic digestion of PLA, PP, and PE and evaluating their biogas production potential as well as the associated chemical and microbiological changes during the process. In this study, the effects of inoculum to substrate ratio (ISR), temperature viz*.,* mesophilic (37 ℃) and thermophilic (55 ℃) conditions and microbiome were studied to understand the feasibility of subjecting the MPs to AD.

## Material and methods

### Substrates

Three different plastics viz*.,* PLA (BioCups, Australia), PP (garden cover), PE (garden cover) were used as substrates. The substrates were shredded to a particle size of 4.75 mm to 2.36 mm by using kitchen blender and sieved through sieves No. 8 and No. 4, respectively.

### Inoculum

Anaerobic digestate from a full-scale biogas plant treating waste activated sludge and primary sludge at 37 ℃ (Queensland Urban Utility, Brisbane) was used as inoculum. The inoculum was sieved through 150 microns sieve to remove large particles and incubated at 37 ℃ and 55 ℃ for 14 days to acclimatise the microorganisms to mesophilic and thermophilic conditions, respectively.

### Batch experiment

The effects of temperature (37 ℃ and 55 ℃) and inoculum to substrate ratio (ISR) of 2, 4, and 6 on the biodegradation of MPs was carried out in 160 ml serum bottles with a working volume of 90 mL (Holliger et al. (2016). Three ISRs were selected to test low to high loading rates and to observe the long lag phase and its effects during the long-term experiment. To each assay, 90 mL inoculum and substrates were added to obtain ISR 2, 4 and 6 on gVS(w/w) basis and distilled water was added to maintain the working volume. Assays without any substrates were also prepared (blank assays) and the methane produced from the inoculum was subtracted from the sample assays (Paulose,Kaparaju 2021). The pH in the assay was maintained at 7.5 using 0.5 M HCl. Upon preparation, the assays were sealed with butyl rubber stoppers and aluminium crimps. Later, assays were flushed with pure N_2_ (99.9%) for two minutes to ensure anaerobic conditions. Two sets of assays were prepared in triplicate and incubated at 37 ℃ and 55 ℃. Experiments were terminated on days of 20, 90 and 148 of the runs. Biogas production volume and composition was monitored regularly. The biogas volume was standardized to normal temperature (25 ℃) and pressure (1 atm), followed by the measurement of carbon dioxide and methane concentrations using the gas chromatograph *Shimadzu GC-2014* with thermal conductivity and packed column of 2.0 m length and 1/16″ outer diameter, 1 mm inner diameter and argon as the carrier (27.5 mL min-1 at 723.8 kPa) (Ketsub 2022). The total biogas and methane production was calculated at standard temperature and pressure (Paulose,Kaparaju 2021). The batch experiment was subjected to kinetic modelling using Modified Gompertz model to determine the theoretical biogas yield and lag phase. The parameters were estimated using nonlinear regression using SPSS 29.

$$B\left( t \right) = B_{0} \cdot \exp \left\{ { - \exp \left[ {\left( {\frac{{R_{\max } *e}}{{B_{0} }}} \right)\left( {\lambda - t} \right) + 1} \right]} \right\}$$

where, B(t) is the theoretical cumulative methane yield (mL Biogas g^−1^VS_added_); B_0_ is the maximum specific methane production potential (mL Biogas g^−1^VS_added_); R_max_ is the maximum specific methane production potential (mL Biogas g^−1^VS_added_); t is the digestion time (days); λ is the lag phase (days) and e = 2.7183.

### Analytical methods

Total solids (TS) and volatile solids (VS) were determined according to Standard Methods (Baxter 2023), pH was monitored by using pH meter (OHAUS Starter 300). The supernatant obtained after centrifugation of digestate at 400 rpm for 15 min (Centurion® C2004), was filtered through 0.45-micron Millipore filter was used for volatile fatty acids (VFA) analysis by using gas chromatography fitted with flame ionization detection (Agilent model 78090A, USA) (Latif et al. 2017). Phosphate and ammonium concentration were analysed by using a flow injection analyser (FIA) Quick Chem 800 (Lachat Instruments, USA) (Latif et al. 2017). Elemental composition (C, H, O, N and S) of substrates were analysed by using Flash*SMART* Elemental Analyzer (Lestari et al. 2023).

### Microbiological analysis

The metagenomic DNA of the digestate was extracted and subjected to amplification of 16S rRNA gene using Bac_SSU_341F (CCTACGGGNGGCWGCAG) and 806WR (GACTACHVGGGTATCTAATCC) primers on a 2 × 300 bp MiSeq sequencing platform to analyse the V3-V4 region (Johani et al. 2018). The sequence obtained was then subjected to QIIME bioinformatic pipeline, followed by taxonomic profiling using Silva SSU database. The obtained feature abundance tables were processed by using MicrobiomeAnalyst (Chong et al. 2020). A total of 1407 singletons were removed, normalized, low count filter of 20% prevalence and low variance filter of 10% were applied to remove low quality features. The diversity indices were calculated at genus level using Welch T-test/ANOVA as the statistical method and the beta diversity represented by Bray–Curtis index using PERMANOVA was also analysed (Chong et al. 2020). The OTU tables were statistically analysed using STAMP2.1.3 and default function of ANOVA with post-hoc Tukey–Kramer (p > 0.05) was applied (Parks et al. 2014). Functional prediction of the microbiome was done using PICRUSt with the KEGG orthologs (Douglas et al. 2018) for obtaining the relative abundance of enzymes and the corresponding pathways using MetaCyc (Caspi et al. 2020). Major 10 pathways were selected through SIMPER analysis using PAST4.16 with Bray–Curtis dissimilarity measure, after pooling all groups to obtain an overall average dissimilarity (Hammer,Harper 2001).

## Results and discussion

### Inoculum and substrates

The characteristics of the inoculum and substrates (PLA, PP and PE) are provided in Table 1. The elemental analysis of the substrates indicated a wide C/N ratio due to high carbon concentration and the absence of nitrogen, unfavourable for the AD process. The wide C/N ratio of bioplastics were reported to hinder the AD process, with co-digestion of food waste reported to improve the methanogenesis (Abraham et al. 2021). The presence of complex backbone linkages, molecule size, crystallinity, hydrophobicity along with additives and stabilisers render them highly resistant to biodegradation (Kubowicz,Booth 2017). These physical, structural and chemical properties of the substrates make them highly recalcitrant in nature and keep the VS content unavailable for biodegradation.

**Table 1:** Chemical properties of the inoculum and substrates

Sample	pH	TS (%)	VS (%)	C (%)	H (%)	N (%)	O (%)	S (%)
PLA	-	99.96	99.33	54.31 ± 0.12	5.59 ± 0.03	0 ± 0	43.68 ± 0.46	0 ± 0
PP	-	98.76	89.80	78.78 ± 0.46	11.52 ± 0.05	0 ± 0	4.46 ± 0.22	0 ± 0
PE	-	98.95	94.01	81.44 ± 0.14	12.23 ± 0.02	0 ± 0	2.63 ± 0.03	0 ± 0
Inoculum (55℃)	7.45	2.76	1.97	-	-	-	-	-
Inoculum (37℃)	7.5	1.18	0.82	-	-	-	-	-

### Biogas production during mesophilic digestion

**Fig. 1:**
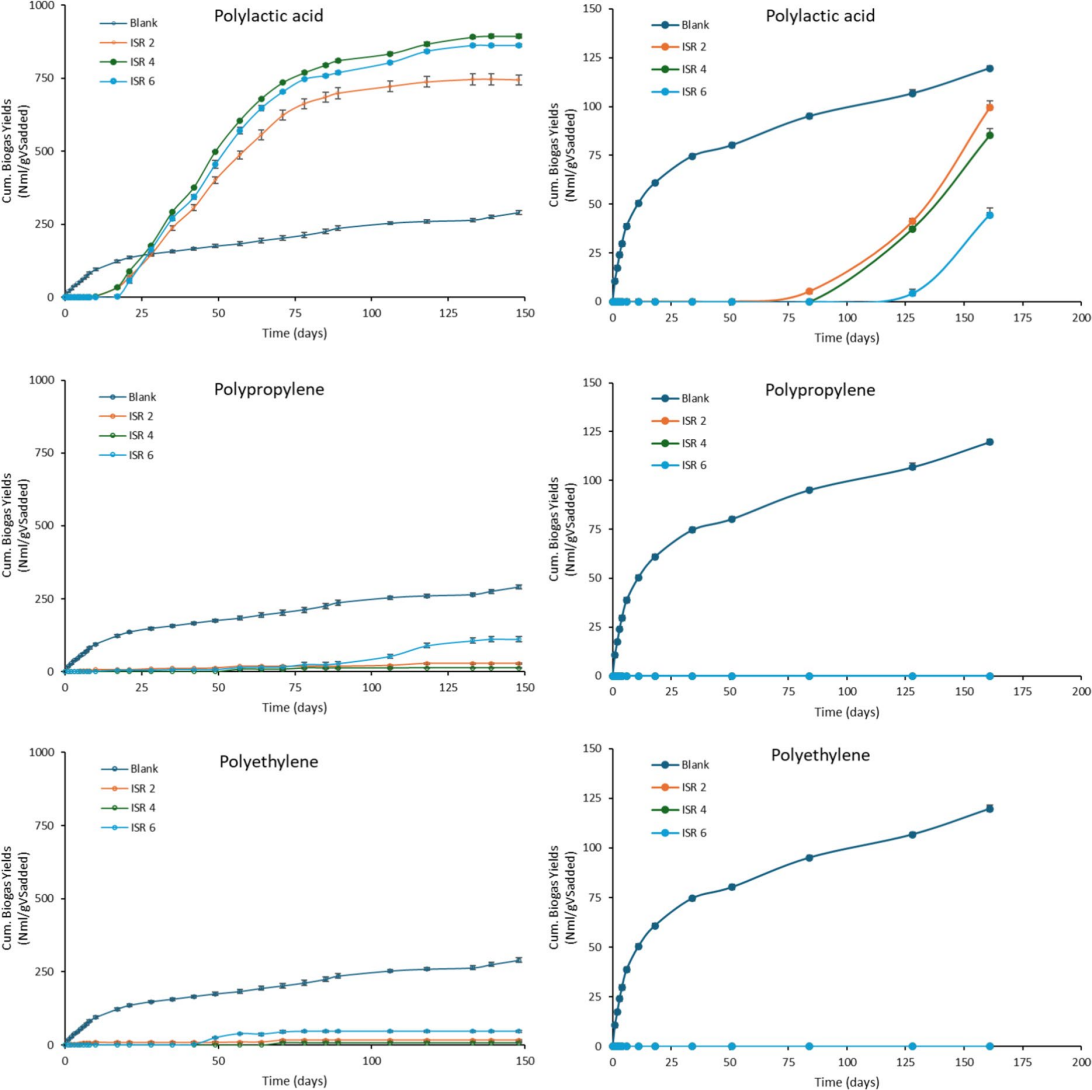
Biogas production under thermophilic (left) and mesophilic (right) anaerobic digestion of PLA, PP and PE for 148 days

The effect of ISR 2, 4 and 6 on biogas production was studied under mesophilic condition (37 ℃) for 161 days. After 161 days, PLA was observed to produce an average of 99.48, 85.13, 44.48 mL biogas/ gVS_added_, at ISR 2, 4 and 6 with ISR 2 showing highest biogas, however not statistically significant (Fig. 1). An early study indicated its inability to degrade PLA after 100 days of incubation at 37 ℃ (Shin et al., 1997). While 80.3 ± 6.1% and 74.7 ± 2% degradation of PLA from two commercial brands under mesophilic AD was observed to occur after 500 days of incubation (Cazaudehore et al. 2023). The current study showed a cumulative biogas production 5.26 mL/gVS_added_, after 84 days of incubation at mesophilic condition at ISR 2, while no production was observed at ISR 4 and 6 at the same period. A long lag phase was observed with the initiation of biogas production after around 70 days in ISR 2 while it extended to 84 and 128 days at ISR 4 and 6 respectively (Fig. 1). Similarly, the mono-digestion of PLA under mesophilic AD for 90 days failed to produce biogas (Vasmara,Marchetti 2016). On the other hand, 155 days of mesophilic AD of PLA showed biodegradation, with 11.1–19.5% residue remaining after the AD process (Falzarano et al. 2025), indicating a better scenario than the current study with a lesser lag phase, however not adaptable for full-scale process due to higher retention time, as indicated in the current study as well. The efficacy of the AD process was observed to be heavily dependent on the nature of PLA blends and structural properties (Falzarano et al. 2025; Lee et al. 2016) as well as the microbial population, its positive interspecific relationships and laboratory adaptation for the process (Chen et al. 2024; Sicchieri et al. 2022). The extremely long lag phase observed in the current study was an indicative of the substrate’s recalcitrancy against the initiation of hydrolysis. The experiment was terminated after 161 days as mesophilic AD was observed to be unsuitable for the degradation of PLA.

The other two substrates PP and PE, failed to generate biogas during the retention period. The presence of PE particles in higher quantities resulted in the inhibition of methane production and caused a shift in microbiome unfavourable for methane production at 37 ℃ (Wei et al. 2019). The inhibition of methane generation was attributed to the smaller sized PE particles adsorbing inhibitors thereby limiting the activities of microorganisms (Akbay et al. 2022). Similarly, the presence of PP resulted in decreased methane yield (Zhang et al. 2022a) and inhibition of methane production under mesophilic conditions (Lim et al. 2018). Inhibition of methanogenesis was reported to be associated with the presence of MPs in waste activated sludge as a result of its impact on methanogens (Zhao et al. 2025). The inhibition of the AD of PP and PE was evident through the increased negative values of cumulative biogas production against the blank over the retention period. Multiple studies have associated process inhibition to the release of ofloxacin, resulting in prolonged lag phases and reduced cumulative biogas production (Xiang et al. 2023), as well as promoting antibiotic resistance and oxidative stress in AD systems (Azizi et al. 2023). The inhibition observed in the current study may be related to similar release of inhibitory substance. The cumulative biogas production for PP and PE was observed to be below the blank indicating the inhibition of the process. The results indicated mesophilic AD to be unsuitable for the degradation of MPs generated from biodegradable and non-biodegradable plastics. The mesophilic AD of PLA indicated a very high hydraulic retention period, while that of PP and PE exhibited process inhibition.

### Biogas production during thermophilic AD

The effect of ISR 2, 4 and 6 on the biodegradation of MPs were studied under thermophilic AD for 148 days. Under thermophilic conditions, PLA was observed to produce a significantly high biogas volume of 894.08 mL/gVS_added_ (CD (0.05) = 27.064) at ISR 4, followed by 862.33 and 744.19 mL/gVS_added_ at ISR 6 and 2 respectively (Fig. 1). The result agreed to a previous study that reported the suitable ISR for AD of PLA to be in between 2.85 and 4, with ISR less than 2 leading to overloading and higher ISR leading to dilution (Cazaudehore et al. 2022b). A study reported 98.96% degradation of untreated PLA under thermophilic AD (58 ℃) for 56 days, while the same was observed to be 0.19% under mesophilic conditions (Vargas et al. 2009), indicating the pivotal role of temperature in PLA degradation. A study conducted by (Bernat et al. 2021) reported that the lag phase of thermophilic AD of PLA to be 10 days, while the lag phase was found to be longer in the current study, with the lowest value observed in ISR 4 (λ = 18.033). Consistent with the current study, a lag phase of 17 days was reported during thermophilic AD of PLA, which was reduced to 12 days through three sequential AD runs, improving the degradation kinetics and microbiological characteristics (Elboghdady et al. 2025). Other studies have reported lag phases of less than one day (Cazaudehore et al. 2022c) and 2.08 and 2.89 days during AD of PLA at 58 ℃ (Cazaudehore et al. 2023). The disparity in lag phase and degradation kinetics are attributed to the properties of the source, concentration, and type of inoculum used (Meghvansi et al. 2022). Adequate acclimation to thermophilic conditions is crucial for optimising AD process efficiency (Shin et al. 2019). The present study further highlights the importance of proper acclimation of inoculum for the thermophilic AD of PLA.

In comparison to PLA, PP and PE generated considerably less biogas under thermophilic conditions, indicating its unsuitability in anaerobic digestion. The thermophilic AD of PP at ISR 6 generated a significantly high average biogas value of 111.64 mL/gVS_added_ compared to 27.79 and 12.88 at ISR 2 and 4 respectively (Fig. 1). A similar scenario was observed in PE degradation with a significantly high mean biogas production of 47.48 mL/gVS_added_ at ISR 6 followed by 16.58 mL/gVS_added_ at ISR 2 and 8.29 mL/gVS_added_ at ISR 4 (Fig. 1). The negligible surface biodegradation of plastics or biogas generation from thermophilic AD of PP and PE was associated with the release of additives and the further utilisation of those compounds by the microorganisms (Belone et al. 2024; Zhang et al. 2022b). Common plasticizers including phthalates present in most of the commercial plastic products were reported to be a potential carbon source for anaerobic microorganisms, while the polymer structure remained intact even after microbial action (Gu 2021). In totality, biodegradation of plastics was observed to be comparatively high under thermophilic conditions (Quecholac-Pina et al. 2020), however not suitable to be adopted for PP and PE microplastic degradation. During the thermophilic AD of PLA, a considerable concentration of methane was also observed to be generated, indicating the prospects of using the same as a potential renewable energy source, if suitable methodologies for improved AD is formulated. However, in the current study, total biogas production was given greater importance to focus on degradation aspects of the substrate over methane generation.

### Methane production by PLA under thermophilic AD

A considerable amount of methane production was also observed during the thermophilic AD of PLA. After reaching a stable biogas production, 717.67 ± 29.87 mL/gVS_added_ with a methane concentration of 53.86 ± 4.23% (n = 70) were observed at ISR 2 after 78 days of AD. At ISR 4, 886.55 ± 11.38 mL/gVS_added_ with a methane concentration of 58.25 ± 1.4% (n = 30) and at ISR 6, 857.38 ± 8.56 mL/gVS_added_ with a methane concentration of 58.4 ± 1.65% (n = 30) were observed in the assays after 118 days of AD. Studies on methane production potential PLA indicated methane generation during the thermophilic AD process, however the substrate to be not suitable for AD due to the extremely high retention period (Cazaudehore et al. 2023), as observed in the current study. The prospects of thermophilic degradation of PLA were suggested to be made efficient by the incorporation of pretreatment strategies to reduce the retention period (Cazaudehore et al. 2022a). Thermophilic AD at ISR between 2.85 and 4 was reported to be optimum for biodegradable plastics (Cazaudehore et al. 2022b) which agrees with the current study considering ISR 4 observed to generate the highest amount of biogas during PLA degradation. However, ISR6 PLA was observed to generate higher cumulative methane yields after 148 days of thermophilic AD with 544.89 NmL CH_4_/gVS_added_ compared to 531.93 and 413.72 NmL CH_4_/gVS_added_ at ISR 4 and 2 respectively (Table S2). The potential use of PLA as a co-substrate with organics as well as using alkali pretreated PLA as mono-substrate for biomethane production was proven by a methane production of 282.7 and 148 L/kg VS_added_ over an HRT of 60 days (Samitthiwetcharong,Chavalparit 2019). However, the present biogas production data indicate that degradation kinetics need to be improved for thermophilic AD to be adopted as an effective strategy for PLA biodegradation.

### Volatile fatty acid profiles and ammonia under thermophilic AD

**Fig. 2:**
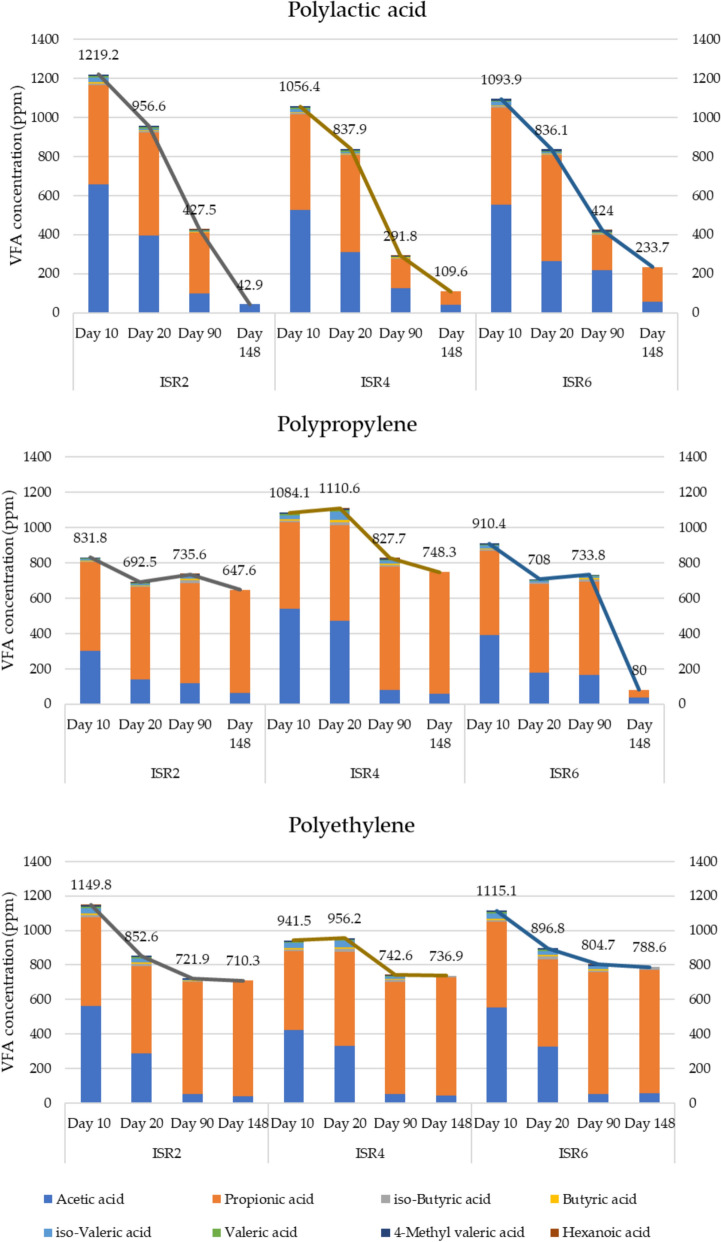
Volatile fatty acids (VFA) utilization during the thermophilic AD of PLA, PP and PE

The volatile fatty acids were analysed for the digestates under thermophilic degradation on days 10, 20, 90 and 148. The VFAs being the intermediates during acidogenesis and acetogenesis, represents the efficiency of the ISR and the overall AD process (Lukitawesa et al. 2020). The efficient utilization of VFAs is essential for AD process, as its accumulation results in acidification and disruption of the process. The efficient VFA utilization occurs as a result of optimum metabolic relation between syntrophic bacteria and methanogens (Zhang et al. 2023). During the thermophilic AD of PLA, a percentage VFA reduction of 96.48, 89.62 and 78.64 were observed at ISR 2, 4 and 6 respectively (Fig. 2). On the contrary, VFA accumulation or inefficient utilization of VFA were observed in the case of PP and PE (Fig. 2). Accumulation of VFAs during the AD process was reported to be associated with low N concentration and wide C/N ratio leading to acidification process (Yasim,Buyong 2023). The concentrations of VFA were reported to be an effective indicator of the AD process with persistence of propionate being associated with its hindrance (Boe et al. 2010). The study also reported propionate accumulation, associated with reactor overloading, was less favourable for VFA degradation and contributed to process failure. During AD, propionic acid persisted in PP and PE degradation, whereas its utilisation in PLA indicated lower stress on the process. The major VFAs during the thermophilic AD were observed to be acetic and propionic acid, while butyric, valeric and hexanoic acid were observed to be present in minor concentrations. During the AD of PLA at ISR 4, the concentration of acetic acid dropped from 527.2 ppm to 41.1 ppm from day 10 to day 148, and that of propionic acid was observed to be 489.5 ppm to 68.5 over the time period. However, considering the percentage concentrations of acetic and propionic acid, the acetic acid content decreased from 49.9% on day 10 to 37.5% on day 148, while that of propionic acid increased from 46.3% to 62.5% over the same period. The inhibitory concentration of propionic acid was reported to be 1000 mg/L, (Sarker et al. 2019), while total VFA exceeding 250–500 mg/L was reported to be detrimental to AD (FAO 1990). In this study, total VFA concentrations were observed to fall below the critical value after 148 days of AD for PLA, whereas it remained above the limits for PP and PE. Previous studies have reported that plasticisers can promote VFA accumulation (Wang et al. 2024), which may have occurred during the AD of PP and PE. An overall efficient VFA utilization during the thermophilic AD of PLA represented an optimal AD process in contrast to its accumulation during the AD of PP and PE.

The analysis of ammoniacal nitrogen (NH_3_) at different stages of the thermophilic AD indicated an increase in its concentration. Studies have indicated the inhibition of methane production due to the presence of ammonia above 1700 mg/L (Yenigün,Demirel 2013). Similarly, concentrations of ammonia near the threshold value that was reported to be detrimental to AD were observed among all the treatments. After 148 days of thermophilic AD, lowest ammonia concentration of 1540 ppm was observed in ISR6 PLA corresponding to the high methane production. While the concentration was observed to fall in between 1670 and 1710 ppm among all the treatments including blank (Table S3). Process inhibition due to NH_3_ accumulation is typically associated with high pH and temperature (Pilarska et al. 2019). However, it was not observed in the present study as the substrates lacked nitrogen, and the observed NH_3_ originated from the inoculum itself, as indicated by the concentration in the blank (Table S3). The production of ammonium nitrogen was reported to be associated with protein degradation (Lukitawesa et al. 2020), however, the lack of its subsequent utilisation was associated with the imbalances in microbial metabolism during the AD process. A study on the impact of polystyrene micro and nano particle in AD also showed high concentration of ammonia which was in turn associated to affect the methanogenic activities (Zhang et al. 2020). The chemical data was considered an indication of unsuitability of thermophilic AD as a prospective method for PP and PE biodegradation, while the reduction of HRT for the same needs to be formulated for PLA biodegradation.

### Microbial population and diversity under thermophilic AD

**Fig. 3:**
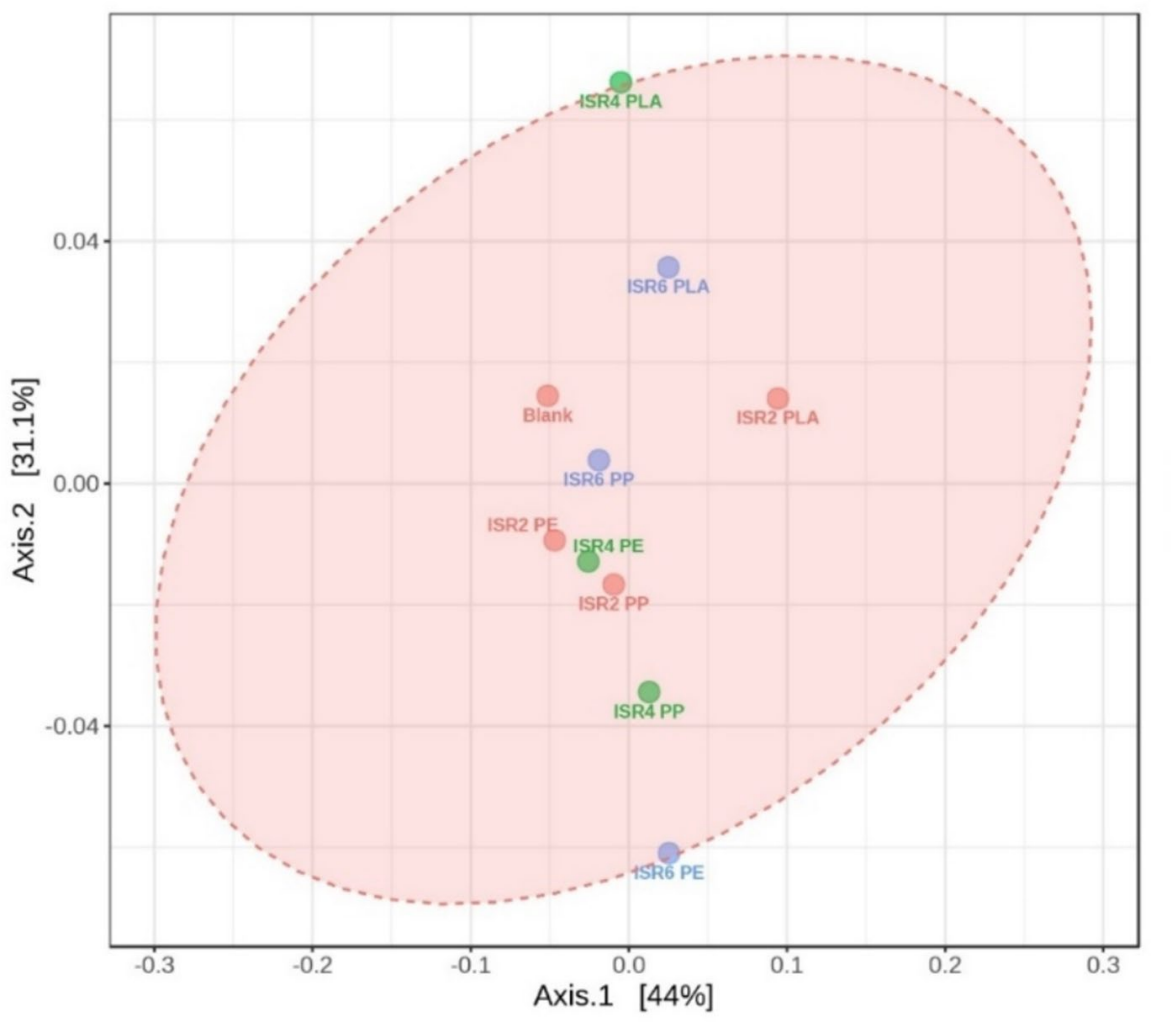
Beta diversity of microorganisms in thermophilic AD at genus level based on Bray–Curtis index

The digestate after thermophilic AD was analysed for the microbial communities using 16S rRNA gene amplicons and the largest library size was observed in ISR4 PP with a total number of reads of 74,339 followed by ISR2 PE and ISR4 PLA with 70,321 and 67,827 respectively, while the highest biogas yield was observed in ISR4 PLA (Table 2). However, the alpha diversity analysis indicated ISR4 PLA to have the highest microbial diversity, while the least was observed in ISR6 PE (Table 2). Similarly, the beta diversity among the samples were analysed through the Bray–Curtis distance (Fig. 3). The highest degree of dissimilarity as indicated by the Euclidean distance on the PCoA matrix was observed for ISR4 PLA. The result indicated ISR4 PLA to harbour a larger diversity of microbiome compared to all the other samples, despite of not having the largest library size. An increased microbial diversity in anaerobic digestion was associated with increased metabolic activity, microbial community resilience and stability (De Vrieze,Verstraete 2016), which was reflected in ISR4 PLA having highest diversity and biogas production. Increased diversity of microorganisms in AD was correlated with improved metabolic function through the metabolic division of labour as well as resource allocation (Zhang et al. 2024c). The high diversity in ISR4 PLA was an indicative of the increased biogas generation, efficient VFA utilisation and increased metabolic activities. The high Shannon index of 4.18 and Simpson index of 0.96 indicated high abundance, richness and evenness in ISR4 PLA compared to other samples. High abundance and evenness were observed to be associated with increased methanogenesis and substrate removal in AD (Werner et al. 2011), in agreement with the results from the current study. Increased species richness in AD was reported to be associated with the functionality of the reactors, suggesting the prevalence of rare syntrophic taxa with low relative abundance to play significant roles in the metabolic activity of AD process (Fujimoto et al. 2019). The increased diversity was in turn correlated with increased functionality, efficacy, resilience, functional diversity and in turn acts against the temporal changes during the AD process (Krohn et al. 2022). Hence, the increased biogas production in ISR4 PLA could be correlated to the high microbial diversity associated with improved metabolic functions.

**Table 2:** Alpha diversity indices and library size of the microbiome under thermophilic AD

Diversity indices	ISR2 PLA	ISR4 PLA	ISR6 PLA	ISR 2 PP	ISR4 PP	ISR6 PP	ISR2 PE	ISR4 PE	ISR6 PE	Blank
Library size	64,389	67,827	57,355	66,223	74,339	65,134	70,321	53,680	63,078	61,057
Simpson	0.959	0.965	0.959	0.959	0.953	0.960	0.958	0.958	0.952	0.960
Shannon	4.046	4.186	4.045	4.021	3.948	4.062	4.061	4.018	3.917	4.084
Chao-1	360	378	342	360	355	355	349	334	344	350

### Bacterial communities at phylum and genus level under thermophilic AD

**Fig. 4:**
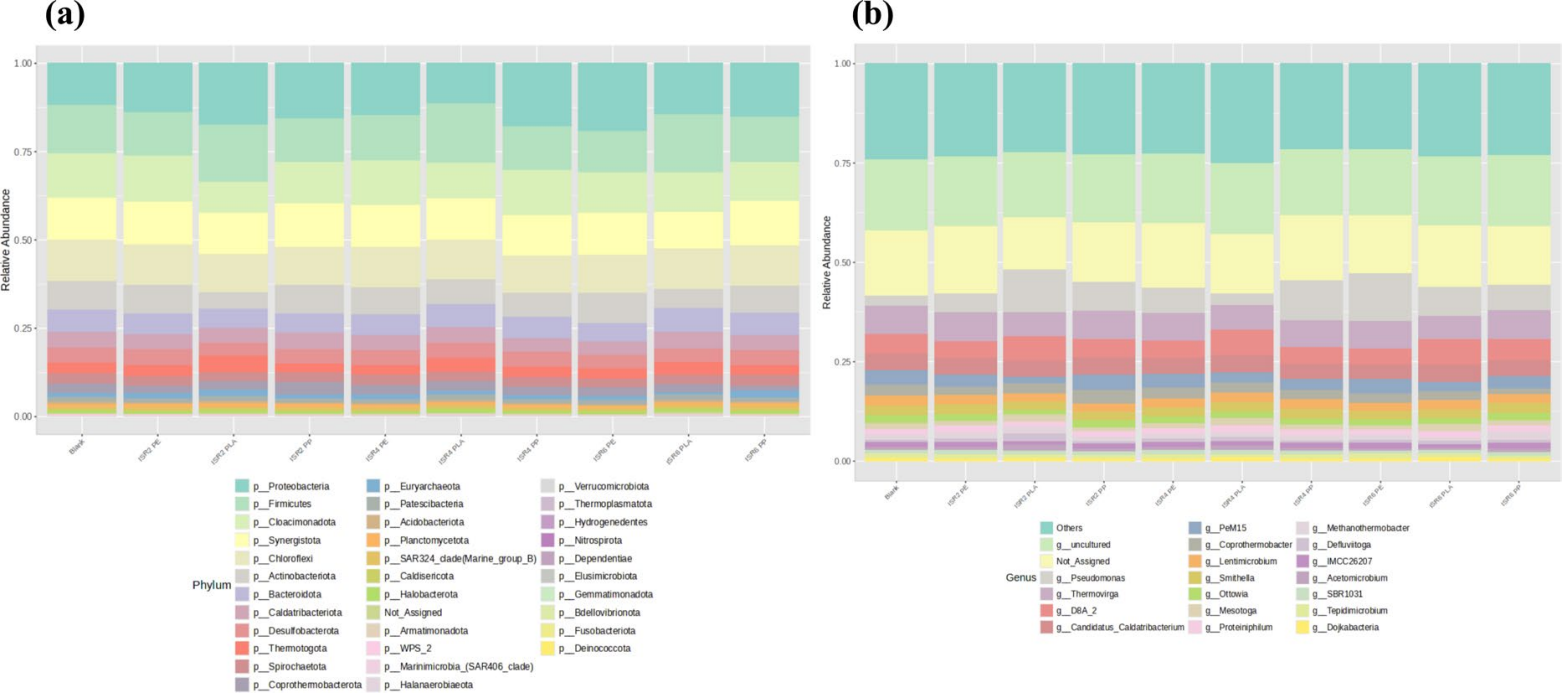
Relative abundance of bacterial phylum **a** and top 20 genera **b** under thermophilic AD

The five most important phyla across all the samples were observed to be Proteobacteria, Firmicutes, Cloacimonadota, Actinobacteriota and Synergistota. Firmicutes were observed to be the most abundant taxa in samples ISR2PLA, ISR4 PLA and ISR6 PLA with 16.21, 16.81 and 16.25 percent relative abundance respectively (Fig. 4a). Proteobacteria was the most abundant phylum in ISR2PLA (17.18%) and across all PP and PE digestates. A high relative abundance of Firmicutes and Proteobacteria were reported to be associated with the thermophilic AD of PLA (Cazaudehore et al. 2022b), which agreed with the current study. The microbiome formed during thermophilic AD of PET, PVC and PLA was observed to be dominated by phylum Firmicutes, Synergistota and Bacteroidota (Zhang et al. 2024a) similar to the population observed in ISR4 PLA. Firmicutes and Bacteroidetes were associated with low organic loading rate and low VFA concentration, indicating conditions favourable for methanogenesis (Chen et al. 2016), as observed in ISR4 PLA. Relative abundance of 6.73 and 6.75 percent of Bacteroidetes was observed in ISR4 and 6 PLA respectively, which was comparatively higher compared to the other samples. Proteobacteria and Actinobacteria, on the other hand was reported to be associated with reduction in methane yields, as they were mostly related with overloading of digestors (Ma et al. 2021). The same study also reported that Proteobacteria mainly being acetogens, and Actinobacteria mainly being acidogens, resulted in VFA accumulation and inhibition of methanogenesis. Propionibacterales, members of phylum Actinobacteria were reported to be associated with production of propionic acid during AD (Harirchi et al. 2022) was observed to be a relevant population among the samples. Phylum Synergistota, observed as the second most abundant phylum in ISR4PLA (11.39%), and among the most abundant phyla across all samples, has been reported to effectively stimulate metabolic activities, enhance the hydrogenotrophic methanogenic pathway, facilitate interspecies electron transfer and utilise AD intermediates (Xu et al. 2024). The population of Cloacimonadota and Actinobacteriota was comparatively high in PP and PE digestates compared to PLA (Fig. 4a). Phylum Cloacimonadota and Desulfobacterota, observed to be present across the samples were reported to be associated with the oxidation of short chain fatty acids, which in turn acted as syntrophs along with methanogens (Dyksma,Gallert 2022). Members of Cloacimonadota were reported to be active syntropic propionate oxidisers (Shi et al. 2024), with active association with acetoclastic and hydrogenotrophic methanogens for H_2_ transfer (Dyksma,Gallert 2022). However, the efficiency of syntrophy is affected by VFA and ammonia concentrations, temperature, with thermophilic being less favourable, substrate composition (Zhang et al. 2023) and the corresponding abundance of *Methanoculleus* sp. and *Methanothermobacter* sp. as syntrophic methanogens (Singh et al. 2023). A combined effect of VFA accumulation, ammonia concentrations, recalcitrant nature of the substrates could have impacted the microbial interactions resulting or vice versa, resulting in an inefficient AD process.

At genus level, all the samples were observed to be dominated by not assigned and unculturable members of the microbiome. Among the identified genera, *Pseudomonas*, D8A-2, *Thermovirga* and Candidatus Caldatribacterium was observed to be abundant in different proportions across the samples (Fig. 4b). A high relative abundance of the unassigned members of Cloacimonadales and Anaerolineaceae were also observed across the samples. The unclassified members of Anaerolineaceae were reported to be acidogens responsible for acetic and propionic acid production (Ao et al. 2021). As discussed earlier regarding the role of Firmicutes in AD, the population of D8A-2 were reported to be associated with syntrophic propionate degradation (Dyksma,Gallert 2022).The genus *Pseudomonas* was reported to be abundant in AD as they were reported to be capable of metabolizing a wide variety of substrates under thermophilic AD (Lin et al. 2016). The relative abundance of the same was observed to be less in uninoculated blank (2.82%) that could be considered due to the deprivation of organic substrates. *Pseudomonas* were also reported to be associated with plastic degradation due to its capacity to secrete hydrolytic enzymes, biofilm formation and surface attachment (Wilkes,Aristilde 2017). The genus *Tepidimicrobium* was stated to play key role in PLA degradation under thermophilic AD (Tseng et al. 2019). Another study on reported *Tepidimicrobium* to play crucial role in the thermophilic AD of PLA due to its lactate utilising capacity (Cazaudehore et al. 2023), the population of which was detected in ISR2 PLA (489 OTUs), however absent in other samples. The same study also reported the relevance of *Coprothermobacter* and Candidatus Caldatribacterium, with the former being a syntroph that supplies hydrogen to hydrogenotrophic archaea and the latter, an acetate oxidizer, both exhibiting syntrophic behaviours with methanogens. Another relatively abundant genus *Thermovirga* was reported to be a representative species for anaerobic digestion stability (An et al. 2024). The genus level analysis of the microbiome indicated the abundance of microorganisms favourable for AD as well as the stability of the system across the samples. However, *Clostridia*, a major acidogen (Harirchi et al. 2022), was found to be dominant in PP and PE digestates (43–49% among Firmicutes), compared to PLA (40% among Firmicutes). Similarly, the abundance of Actinobacteriota in PLA was observed to be 5%, 7% and 6% in ISR 2, 4 and 6, respectively, whereas in PP and PE digestates, it ranged from 8–9%. The higher abundance of acidogens in PP and PE digestates may be a contributing factor to the observed VFA accumulation.

Overall, the microbial community analysis highlighted the importance of Firmicutes and Proteobacteria, which were observed to be abundant, and were the major hydrolytic members during the AD process (Zhang et al. 2024b). The primary VFAs observed were acetic and propionic acids, and a a notably high population of acidogens were detected. Propionibacterales (Actinobacteria), associated with propionate production, members of Clostridia, linked to acetic, propionic and butyric acid production, while *Moorella*, associated with acetic acid production (Harirchi et al. 2022)were abundant across all the samples, indicating the key acidogens present. Members of phylum Synergistota and Cloacimonadota likely functioned as major propionate- and VFA-utilising bacteria, facilitating H2 transfer to methanogens and supporting hydrogenotrophic methanogenesis (Dyksma,Gallert 2022; Shi et al. 2024).

### Archaebacterial population and diversity under thermophilic AD

**Fig. 5:**
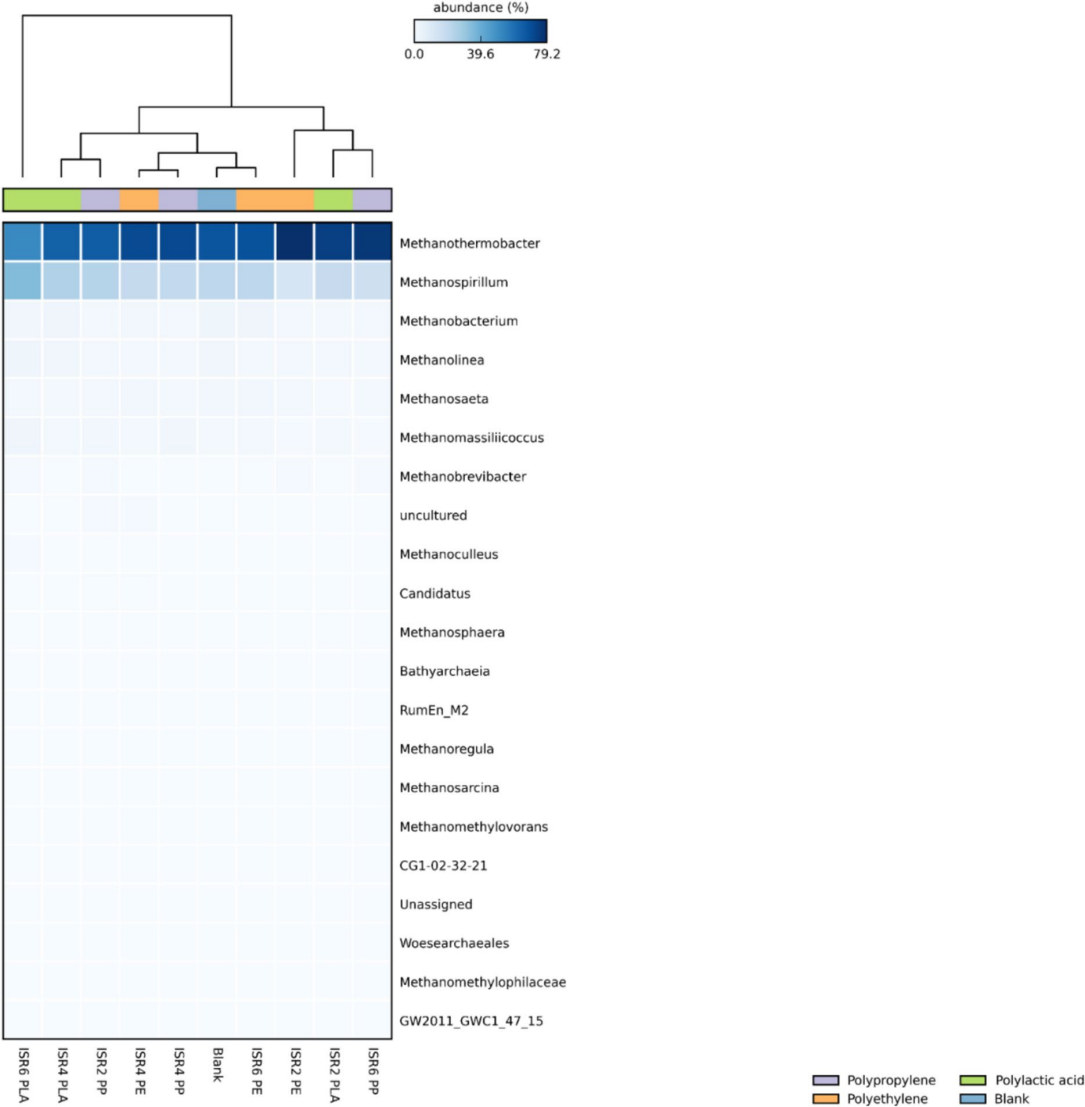
Relative abundance of archaea across the samples after thermophilic AD (*p* > 0.05)

The largest population of total archaea was observed in ISR6 PP with a total number of reads of 1746, while the least number was observed in ISR6 PLA with a total number of reads of 747. On the contrary, the largest archaebacterial diversity was observed in ISR6 PLA with a Shannon index of 1.202 and Simpson index of 0.604 (Table S4) indicating increased species richness, abundance and evenness of archaeal population, compared to the other samples. As discussed previously, diversity to be positively linked with metabolic functions, resilience and stability was reflected in the increased methanogenesis in ISR6 PLA with highest value of cumulative methane production after 148 days of thermophilic AD. The most abundant methanogen was observed to be *Methanothermobacter*, followed by *Methanospirillum* and *Methanobacterium* (Fig. 5). The genus *Methanothermobacter* was reported to be a hydrogenotrophic methanogen that prefers a propionic rich environment (Tian et al. 2015). A syntrophic relationship with *Clostrdia*, *Synergista* and *Thermotogae* (Mu et al. 2025) and *Coprothermobacter* and DTU014 (Jin et al. 2022), was reported to be essential for the thermophilic AD of PLA, with these populations observed across the samples. As the microbial analysis was done at the end of the experiment after 148 days, the increased relative abundance of *Methanothermobacter* in ISR6 PP could be associated with the rapid surge in biogas production (Fig. 1) and the VFA utilisation (Fig. 2) after 90 days of inoculation, which was associated with the probable degradation of additives. However, a similarly high population of *Methanothermobacter* was observed in ISR2PE, yet no increase in methanogenesis was noticed. This discrepancy may be associated with the increased abundance of Synergistia, Thermotogae and the secondary methanogen *Methanospirillum* in ISR6PP. *Coprothermobacter*, a proteolytic bacteria reported to be a strict syntroph with *Methanothermobacter* (Gagliano et al. 2015) was observed across the samples.

The second most abundant methanogen *Methanospirillum* was found to be a hydrogenotrophic methanogen in agro-industrial waste AD (Jain et al. 2021), especially under ammonia rich conditions (Shi et al. 2018) and the third-most abundant methanogen, *Methanobacterium* was also observed to follow hydrogenotrophic pathway (Cai et al. 2016). However, *Methanobacterium* was also reported to utilise CO_2_ and H_2_ converted by acetate oxidizing rod through non-acetoclastic acetate utilising methanogenesis (Sun et al. 2021; Zinder 1990). Major acetate oxidizers were reported to be associated with phylum Firmicutes (Pan et al. 2021) and hence the corresponding abundance of the methanogen. The CO_2_ and H_2_ supplied by the syntroph, in particular *Tepidimicrobium* was observed to be utilised by *Methanothermobacter* for methanogenesis of PLA under thermophilic AD (Tseng et al. 2019). Hence, the archaeal population of the thermophilic AD of PLA, PP and PE indicated the dominance of hydrogenotrophic methane production and minor presence of acetoclastic methanogenesis after the metabolization of polymers by the previously mentioned hydrolytic microorganisms. The reduction on biogas production as a result of the inhibition of *Methanosaeta*, an abundant acetoclastic methanogen was observed to be association with MP mediated process inhibition in AD (Zhao et al. 2025). The population of these organisms across the samples were observed to be greatly reduced, indicating a shift in the archaeal community that was not conducive to methanogenesis.

### Metabolic profiles of thermophilic AD

**Fig. 6:**
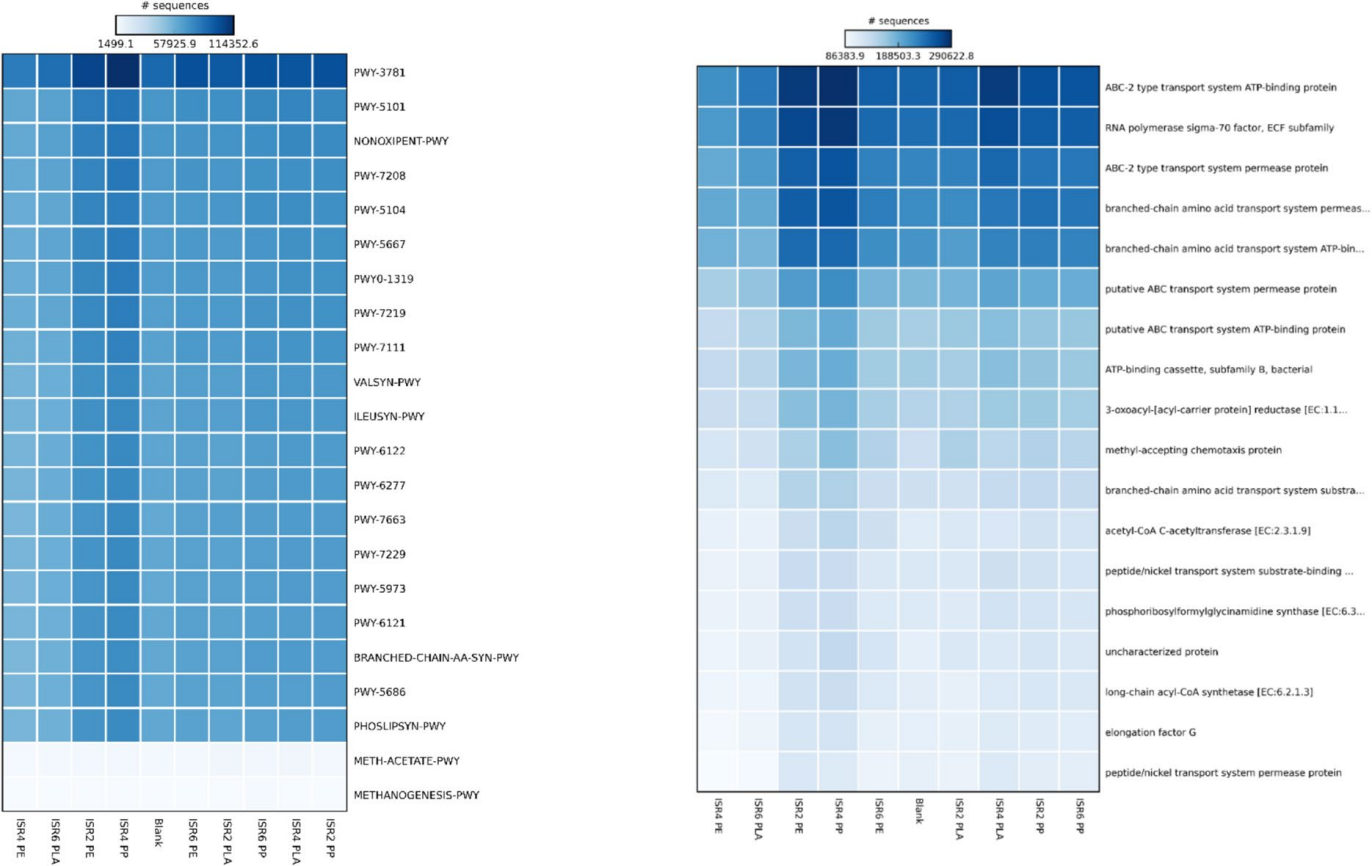
Top 20 metabolic pathways and enzymes corresponding to the 20 most abundant KO numbers (*p* > 0.05)

The study of map pathways by analysing the top 20 abundant KO numbers against KEGG database produced matches for quorum sensing, fatty acid metabolism, fatty acid biosynthesis, metabolic pathways, secondary metabolites biosynthesis and microbial metabolism in diverse environments being a few relevant ones. Metabolic predictions of the thermophilic AD microbiome indicated an abundance of aerobic respiration pathway in PP and PE, possibly due to the reactive oxygen species (ROS) production, along with increased fatty acid metabolism, microbial adaptation and carbon metabolism across all samples. The major enzymes in terms of its relative abundance across the samples indicated PWY-3781, PWY-5101, PW-5104, NONOXIPENT-PWY, PWY-7208, PWY-7111, PWY-7219, PWY-5667, PWY0-1319 and ILEUSYN-PWY (Fig. 6). An average overall dissimilarity of 6.273 was measured and a significance difference in the relative abundance of metabolic functions were not observed across the samples. The most abundant pathway observed, PWY-3781 aerobic respiration 1 (cytorchrome c) often associated with reactive oxygen species (ROS) detoxification (Ren et al. 2024) was abundant in PP and PE degradation. A study reported the presence polyamide (PA) MPs in AD to be associated with ROS generation (Xiang et al. 2024), while AD exposed to PE MPs resulted in reduced microbial cell viability by 7.6–15.4% (Wei et al. 2019). The ROS stress was reported to be associated with the activity of *Pseudomonas* (Li et al. 2024) which could have affected the functionality of obligate anaerobes like *Coprothermobacter* and Synergistota that played crucial role in syntrophic relationships. Also, the high propionate concentration, VFA accumulation and its increased concentration in AD was reported to trigger ROS stress, while the population of *Syntrophomonas* and *Syntrophobacter* were found to be relevant in alleviating the stress (Yan et al. 2023), whose population was observed across the samples. As discussed earlier, PP and PE associated with VFA accumulation with high propionate concentration could be correlated with the abundance of PWY-3781 in the order PE > PP > PLA as an adaptive mechanism.

The presence of active metabolism in terms of amino acid synthesis as indicated by the abundant of L-isoleucine biosynthesis (PWY-5101, ILEUSYN-PWY), pentose phosphate pathway (NONXIPENT-PWY), adenosine ribonucleotides *de* novo biosynthesis (PWY-7219), CDP-diacyglycerol biosynthesis 1 (PWY-5667, PWY-1319), which utilises fatty acids through esterification, pyruvate fermentation to isobutanol (PWY-7111) and pyrimidine nucleobases salvage (PWY-7208) (Caspi et al. 2020). The presence of LACTOSECAT-PWY that corresponds to the cleavage of lactose to glucose and galactose was observed, corresponding to the abundance of Lactobacillales (Smid et al. 2005) across all the samples. KO numbers corresponding to lactate dehydrogenase (K00782, K00016, K00102, K18929, K03778) that converts pyruvate to lactate and vice versa was observed to be abundant in ISR4 PLA compared to other samples, indicating active lactate metabolism. The presence of K00496 corresponding to alkane 1-monooxygenase was observed to be abundant in PP and PE degradation compared to PLA was reported to be associated with low molecular weight PE degradation (Jeon,Kim 2016). The analysis of pathways, KEGG orthologs and the corresponding function correlated with the abundance of bacterial taxa related to the functions was an indicative of the formation of plastisphere microorganisms in relation to the MPs present.

## Conclusion

Among the three polymer types tested, only PLA was observed to be degraded under thermophilic AD and highest cumulative biogas production was obtained at ISR 4. The comparative efficiency of PLA degradation was reflected in the VFA utilisation observed, compared to the VFA accumulation associated with PP and PE. The results indicated thermophilic AD to be a prospective degradation strategy for PLA. Mesophilic AD was found to require an extremely long period for the initiation of hydrolysis, and process inhibition was observed with PP and PE. Also, the contrary, the mono-digestion of PP and PE was unsuitable for thermophilic AD and while inhibition was observed under mesophilic AD. The microbiome analysis of the thermophilic AD indicated the presence of microbiome favourable for methanogenesis in PLA while it shifted against methanogenesis in PP and PE, with hydrogenotrophic methanogenesis being the major pathway. The functional profiling indicated the abundance of pathways associated with aerobic respiration, fatty acid metabolism, fatty acid biosynthesis, metabolic pathways, secondary metabolites biosynthesis. The study showed the prospect of the suitability of thermophilic AD in PLA biodegradation considering suitable pretreatment strategies, while the same to be unsuitable for the degradation of PP and PE. Further studies need to be conducted to analyse the presence of PLA residues and degradation intermediates to determine the suitability of its release into environment.

## Supplementary Information

Below is the link to the electronic supplementary material.

Supplementary file1 (DOCX 32 KB)

## Data Availability

No datasets were generated or analysed during the current study.
