# Supplementary material for: Thermophilic anaerobic digestion of polylactic acid, polyethylene and polypropylene microplastics: effect of inoculum-substrate ratio and microbiome
Source: Biodegradation. 2025 Sep 30;36(5):95. doi: 10.1007/s10532-025-10186-6 (PMC12484357; doi:10.1007/s10532-025-10186-6)
Supplement: Supplementary file 1 — Supplementary file1 (DOCX 32 KB) [file 10532_2025_10186_MOESM1_ESM.docx]

**Table S1** Cumulative methane production under mesophilic AD (NmL CH_4_/g VS added)

| Days |  | PLA | | | PP | | | PE | | |
| --- | --- | --- | --- | --- | --- | --- | --- | --- | --- | --- |
|  | **Blank** | **ISR 2** | **ISR 4** | **ISR 6** | **ISR 2** | **ISR 4** | **ISR 6** | **ISR 2** | **ISR 4** | **ISR 6** |
| 0 | 0.00 | 0.00 | 0.00 | 0.00 | 0.00 | 0.00 | 0.00 | 0.00 | 0.00 | 0.00 |
| 1 | 0.97 | 0.00 | 0.00 | 0.00 | 0.00 | 0.00 | 0.00 | 0.00 | 0.00 | 0.00 |
| 2 | 2.10 | 0.00 | 0.00 | 0.00 | 0.00 | 0.00 | 0.00 | 0.00 | 0.00 | 0.00 |
| 3 | 3.51 | 0.00 | 0.00 | 0.00 | 0.00 | 0.00 | 0.00 | 0.00 | 0.00 | 0.00 |
| 4 | 5.06 | 0.00 | 0.00 | 0.00 | 0.00 | 0.00 | 0.00 | 0.00 | 0.00 | 0.00 |
| 6 | 8.29 | 0.00 | 0.00 | 0.00 | 0.00 | 0.00 | 0.00 | 0.00 | 0.00 | 0.00 |
| 11 | 13.28 | 0.00 | 0.00 | 0.00 | 0.00 | 0.00 | 0.00 | 0.00 | 0.00 | 0.00 |
| 18 | 19.09 | 0.00 | 0.00 | 0.00 | 0.00 | 0.00 | 0.00 | 0.00 | 0.00 | 0.00 |
| 34 | 28.56 | 0.00 | 0.00 | 0.00 | 0.00 | 0.00 | 0.00 | 0.00 | 0.00 | 0.00 |
| 51 | 32.31 | 0.00 | 0.00 | 0.00 | 0.00 | 0.00 | 0.00 | 0.00 | 0.00 | 0.00 |
| 84 | 43.16 | 6.79 | 3.31 | 0.00 | 0.00 | 0.00 | 0.00 | 0.00 | 0.00 | 0.00 |
| 128 | 52.13 | 30.21 | 30.71 | 11.49 | 0.00 | 0.00 | 0.00 | 0.00 | 0.00 | 0.00 |
| 161 | 62.14 | 67.32 | 61.09 | 37.11 | 0.00 | 0.00 | 0.00 | 0.00 | 0.00 | 0.00 |

**Table S2** Cumulative methane production under thermophilic AD (NmL CH_4_/g VS added

| Days |  | PLA | | | PP | | | PE | | |
| --- | --- | --- | --- | --- | --- | --- | --- | --- | --- | --- |
|  | **Blank** | **ISR 2** | **ISR 4** | **ISR 6** | **ISR 2** | **ISR 4** | **ISR 6** | **ISR 2** | **ISR 4** | **ISR 6** |
| 0 | 0.00 | 0.00 | 0.00 | 0.00 | 0.00 | 0.00 | 0.00 | 0.00 | 0.00 | 0.00 |
| 1 | 0.21 | 0.00 | 0.00 | 0.00 | 0.00 | 0.00 | 0.00 | 0.00 | 0.00 | 0.00 |
| 2 | 0.54 | 0.00 | 0.00 | 0.00 | 0.00 | 0.00 | 0.00 | 0.00 | 0.10 | 0.35 |
| 3 | 1.25 | 0.00 | 0.00 | 0.00 | 0.00 | 0.00 | 0.00 | 0.00 | 0.15 | 0.35 |
| 4 | 1.93 | 0.00 | 0.00 | 0.00 | 0.14 | 0.00 | 0.00 | 0.00 | 0.15 | 0.35 |
| 5 | 2.81 | 0.00 | 0.00 | 0.00 | 0.91 | 0.00 | 0.00 | 0.21 | 0.20 | 0.56 |
| 6 | 3.81 | 0.00 | 0.00 | 0.00 | 1.50 | 0.00 | 0.03 | 0.37 | 0.35 | 0.56 |
| 7 | 5.02 | 0.00 | 0.00 | 0.00 | 3.04 | 0.00 | 1.24 | 0.49 | 0.35 | 0.56 |
| 8 | 6.66 | 0.18 | 1.22 | 0.00 | 5.02 | 0.00 | 2.35 | 0.77 | 0.35 | 0.56 |
| 10 | 9.28 | 2.87 | 5.74 | 0.04 | 7.47 | 0.00 | 9.01 | 2.40 | 0.35 | 0.56 |
| 17 | 17.84 | 20.97 | 27.24 | 15.08 | 9.24 | 0.00 | 19.17 | 2.40 | 0.35 | 0.56 |
| 21 | 21.68 | 44.69 | 65.08 | 54.31 | 11.15 | 0.00 | 19.60 | 2.40 | 0.35 | 0.56 |
| 28 | 25.46 | 93.45 | 122.26 | 124.18 | 13.67 | 0.00 | 19.60 | 2.40 | 0.35 | 0.56 |
| 35 | 28.29 | 150.69 | 195.59 | 194.44 | 14.42 | 0.75 | 26.09 | 2.40 | 0.35 | 0.56 |
| 42 | 31.54 | 187.39 | 246.45 | 240.25 | 15.01 | 0.75 | 26.09 | 2.40 | 0.35 | 0.84 |
| 49 | 34.57 | 238.17 | 322.23 | 309.76 | 17.66 | 4.40 | 26.09 | 2.40 | 0.35 | 14.21 |
| 57 | 37.46 | 285.33 | 385.90 | 385.25 | 20.16 | 14.65 | 33.67 | 3.04 | 0.37 | 24.95 |
| 64 | 41.37 | 320.80 | 426.98 | 431.83 | 20.16 | 14.65 | 34.46 | 3.04 | 1.22 | 24.95 |
| 71 | 44.37 | 354.86 | 454.19 | 462.78 | 20.16 | 14.65 | 35.01 | 6.57 | 6.07 | 28.98 |
| 78 | 47.73 | 372.84 | 470.71 | 485.83 | 20.16 | 17.07 | 38.86 | 6.57 | 6.07 | 30.32 |
| 85 | 52.25 | 382.63 | 483.10 | 489.43 | 20.16 | 17.07 | 38.86 | 6.57 | 6.07 | 30.32 |
| 89 | 56.12 | 387.28 | 487.73 | 492.26 | 20.35 | 17.07 | 40.71 | 6.57 | 6.07 | 30.32 |
| 106 | 62.51 | 400.50 | 499.20 | 511.42 | 22.51 | 17.07 | 62.46 | 6.57 | 6.07 | 30.32 |
| 118 | 64.81 | 409.18 | 517.89 | 534.05 | 25.41 | 17.07 | 89.80 | 6.57 | 6.07 | 30.32 |
| 133 | 66.45 | 413.72 | 531.93 | 544.89 | 25.41 | 17.07 | 104.15 | 6.57 | 6.07 | 30.32 |
| 139 | 70.59 | 413.72 | 531.93 | 544.89 | 25.41 | 17.07 | 110.04 | 6.57 | 6.07 | 30.32 |
| 148 | 76.33 | 413.72 | 531.93 | 544.89 | 25.41 | 17.07 | 110.04 | 6.57 | 6.07 | 30.32 |

**Table S3** Nitrite, nitrate, ammonia and phosphate concentrations during thermophilic AD

|  |  | NO_2_-N (ppm) | NO_3_-N (ppm) | NH_3_-N (ppm) | PO_4_-P (ppm) |
| --- | --- | --- | --- | --- | --- |
| PLA | ISR2 | 1 | 78 | 1700 | 162 |
|  | ISR4 | 1 | 79 | 1670 | 159 |
|  | ISR6 | 1 | 79 | 1540 | 145 |
| PP | ISR2 | 1 | 79 | 1700 | 167 |
|  | ISR4 | 1 | 79 | 1700 | 162 |
|  | ISR6 | 0 | 79 | 1650 | 159 |
| PE | ISR2 | 1 | 79 | 1670 | 160 |
|  | ISR4 | 1 | 78 | 1700 | 160 |
|  | ISR6 | 1 | 79 | 1690 | 163 |
|  | Blank | 1 | 78 | 1710 | 169 |

**Table S4** Alpha diversity indices and library size of archaebacteria under thermophilic AD

| Diversity indices | PLA | | | PP | | | PE | | |
| --- | --- | --- | --- | --- | --- | --- | --- | --- | --- |
|  | **ISR 2** | **ISR2 4** | **ISR 6** | **ISR 2** | **ISR 4** | **ISR 6** | **ISR 2** | **ISR 4** | **ISR 6** |
| Library size | 1443 | 1253 | 747 | 1056 | 1252 | 1764 | 1440 | 857 | 1042 |
| Simpson | 0.4072 | 0.5207 | 0.6045 | 0.5085 | 0.4498 | 0.3893 | 0.355 | 0.4541 | 0.4764 |
| Shannon | 0.8047 | 1.05 | 1.202 | 1.044 | 0.8955 | 0.8271 | 0.7771 | 0.9335 | 0.9521 |
| Chao-1 | 7 | 10 | 9 | 9 | 7 | 10 | 8 | 8 | 7 |
